# Complete genome sequence of antibiotic-producing *Streptomyces* sp. HBERC⁃20821, isolated from Wawushan Hill, Sichuan Province, China

**DOI:** 10.1128/mra.00804-24

**Published:** 2024-09-16

**Authors:** Yifan Zhang, Zhongyi Wan, Ling Chen, Lei Zhu, Yimin Qiu, Wei Chen, Xiaoyan Liu, Yong Min

**Affiliations:** 1 Hubei Biopesticide Engineering Research Center, Hubei Academy of Agricultural Sciences, Wuhan, China; 2 School of Life and Health Sciences, Hubei University of Technology, Wuhan, China; University of Maryland School of Medicine, Baltimore, Maryland, USA

**Keywords:** complete genome, genetic manipulation, *Streptomyces *sp.

## Abstract

The complete genome of a *Streptomyces* capable of producing multiple antibiotics was sequenced. Strain HBERC-20821 was isolated from a soil sample collected at Wawushan Hill, Sichuan Province, China. Genomic information will facilitate our systematic genetic manipulation of the strain at the gene level, enhancing its antibiotic production.

## ANNOUNCEMENT

Strain HBERC-20821, identified as a *Streptomyces* species, generates various antifungal secondary metabolites like novonestmycin, with its fermentation broth demonstrating efficacy in field control of rice sheath blight, cucumber gray mold, and tea anthracnose (1). It was isolated from a soil sample collected at Wawushan Hill, Sichuan province, China (29° 38' 34.4" N, 102° 55' 55.99" E), and the concentration in the natural soil sample is 2 × 10⁻⁴ CFU/mL. Soil suspensions were serially diluted with sterile water and spread onto solid culture media supplemented with antibiotics (cycloheximide, terbinafin, and nalidixic acid) to isolate single colonies. Then, we purified the colonies on the ISP-2 media. The genomic DNA of strain HBERC-20821 was extracted using the Genomic-tip 100 /G DNA kit (Qiagen).

The genome of HBERC-20821 was sequenced using a combined Illumina/Oxford Nanopore sequencing approach. First, sequencing was conducted using the Illumina NovaSeq sequencing platform. The libraries were quantified using PicoGreen, and the PCR-enriched fragments were subjected to quality control using the Agilent Bioanalyzer 2100 to verify the fragment size and distribution of the DNA libraries. Quality control of the data was performed using fastp (2) (https://github.com/OpenGene/fastp). Filtering resulted in 21,910,400 high-quality reads, which was 98.42% of the total reads sequenced. The average size of the Illumina NovaSeq sequencing paired-end (PE) library is 400 bp, prepared using the TruSeq DNA Sample Prep Kit, with sequencing read lengths of 150 bp. Furthermore, the library was sequenced on the Oxford Nanopore PromethION 48 platform without DNA shearing or size selection. Sequencing was performed using the SQK-LSK109 ligation kit, and libraries were sequenced with an R9.4.1 flow cell. The final quality statistics included 1,021,862 reads. Flye and Unicycler software were used to assemble the data obtained by Nanopore platform sequencing. Subsequently, we selected the assembly results from the software that performed best to generate a complete sequence. Finally, high-quality Illumina data were used to correct an ONT assembly using Pilon (3). A linear, gapless chromosomal genome was assembled without telomeres, and the size of the species genome closely matched that of previously published genomes, thereby confirming the completeness of the genome assembly. The assembly quality of the final complete sequence was evaluated, revealing a genome sequence length of 6,370,257 bp and a GC content of 73.1%, with genome coverage of 709×.

Using the GeneMarkS software, 5,323 protein-coding genes were predicted. The gene-encoded protein sequences were aligned with the protein sequences in the eggNOG (COG) database using Diamond BLASTP (2). Additional 53 non-coding RNA genes were predicted by comparison with the Rfam database. Utilizing CRISPR finder, 16 CRISPR structures were predicted (4, 5), thus laying the foundation for subsequent gene knockout experiments (6–8). Moreover, antiSMASH v6.0 (9) analysis of the strain predicted 23 gene clusters for biosynthesis of secondary metabolites.

The genome of HBERC-20821 has an average nucleotide identity (ANI) of 99.1% with *Streptomyces* sp. Z26 (GenBank accession number RCHV00000000), as determined using the ANI Calculator (https://www.ezbiocloud.net) (10). Therefore, HBERC-20821 was identified as a *Streptomyces* species isolate.

## Data Availability

The raw sequences are available under Sequence Read Archive (SRA) accession numbers SRR29689291 (Illumina) and SRR29699762 (ONT), and the genome assembly is available in GenBank under accession number CP160458.

## References

[1] Wan Z, Fang W, Shi L, Wang K, Zhang Y, Zhang Z, Wu Z, Yang Z, Gu Y. 2015. Novonestmycins A and B, two new 32-membered bioactive macrolides from Streptomyces phytohabitans HBERC-20821. J Antibiot 68:185–190. doi:10.1038/ja.2014.123 25204346

[2] Buchfink B, Xie C, Huson DH. 2015. Fast and sensitive protein alignment using DIAMOND. Nat Methods 12:59–60. doi:10.1038/nmeth.3176 25402007

[3] Walker BJ, Abeel T, Shea T, Priest M, Abouelliel A, Sakthikumar S, Cuomo CA, Zeng Q, Wortman J, Young SK, Earl AM. 2014. Pilon: an integrated tool for comprehensive microbial variant detection and genome assembly improvement. PLoS One 9:e112963. doi:10.1371/journal.pone.0112963 25409509 PMC4237348

[4] Grissa I, Vergnaud G, Pourcel C. 2007. CRISPRFinder: a web tool to identify clustered regularly interspaced short palindromic repeats. Nucleic Acids Res 35:W52–W57. doi:10.1093/nar/gkm360 17537822 PMC1933234

[5] Symeonidi E, Regalado J, Schwab R, Weigel D. 2021. CRISPR-finder: a high throughput and cost-effective method to identify successfully edited Arabidopsis thaliana individuals. Quant Plant Biol 2:e1. doi:10.1017/qpb.2020.6 37077216 PMC10095899

[6] Lee Y, Hwang S, Kim W, Kim JH, Palsson BO, Cho B-K. 2024. CRISPR-aided genome engineering for secondary metabolite biosynthesis in Streptomyces. J Ind Microbiol Biotechnol 51:kuae009. doi:10.1093/jimb/kuae009 38439699 PMC10949845

[7] Hua HM, Xu JF, Huang XS, Zimin AA, Wang WF, Lu YH. 2024. Low-toxicity and high-efficiency Streptomyces genome editing tool based on the miniature type V-F CRISPR/Cas nuclease AsCas12f1. J Agric Food Chem 72:5358–5367. doi:10.1021/acs.jafc.3c09101 38427033

[8] Tan LL, Heng E, Leong CY, Ng V, Yang LK, Seow DCS, Koduru L, Kanagasundaram Y, Ng SB, Peh G, Lim YH, Wong FT. 2024. Application of Cas12j for Streptomyces editing. Biomolecules 14:486. doi:10.3390/biom14040486 38672502 PMC11048056

[9] Blin K, Shaw S, Kloosterman AM, Charlop-Powers Z, van Wezel GP, Medema MH, Weber T. 2021. antiSMASH 6.0: improving cluster detection and comparison capabilities. Nucleic Acids Res 49:W29–W35. doi:10.1093/nar/gkab335 33978755 PMC8262755

[10] Buchmann A, Cano-Prieto C, Nafis A, Barakate M, Baz M, Hassani L, Ortlieb N, Niedermeyer THJ, Gross H. 2019. Draft genome sequence of the novonestmycin-producing strain Streptomyces sp. Z26, isolated from potato rhizosphere in Morocco. Microbiol Resour Announc 8:10–1128. doi:10.1128/MRA.01514-18 PMC6318375 30637404

